# Outcomes from the medication assisted treatment pilot program for adults with opioid use disorders in rural Colorado

**DOI:** 10.1186/s13011-021-00424-4

**Published:** 2022-01-03

**Authors:** Claudia R. Amura, Tanya R. Sorrell, Mary Weber, Andrea Alvarez, Nancy Beste, Ursula Hollins, Paul F. Cook

**Affiliations:** 1 University of Colorado College of Nursing, 13120 E. 19th Ave, Aurora, CO 80045 USA; 2 Present address: Rush University College of Medicine, Chicago, IL 60612 USA; 3 Present Address: Great Lakes Region NIH NIDA CCTN, Chicago, USA; 4 Health Solutions Inc, 41 Montebello Rd., Pueblo, CO 81001 USA; 5 Mountain Medical: Road to Recovery, PO Box 773705, Steamboat Springs, CO 80477 USA; 6 Colorado Treatment Services, 511 W. 29th St. Pueblo, CO 91008 USA Colorado Treatment Services, Pueblo, CO 91008 USA

**Keywords:** Medication-assisted treatment for opioid use disorder, Pain, Rural, Community settings, Patient centered outcomes

## Abstract

**Background:**

As Colorado ranked among the top nationally in non-medical use of opioids, a pilot medication for opioid use disorder (MOUD) program was developed to increase the number of NPs and PAs providing MOUD in order to bring this evidence- based treatment to 2 counties showing disproportionally high opioid overdose deaths. Over the first 18 months, the MOUD Pilot Program led to 15 new health care providers receiving MOUD waiver training and 1005 patients receiving MOUD from the 3 participating organizations. Here we evaluate patient centered clinical and functional outcomes of the pilot MOUD program implemented in 2 rural counties severely affected by the opioid crisis.

**Methods:**

Under state-funded law (Colorado Senate Bill 17–074), three rural agencies submitted de-identified patient-level data at baseline (*N* = 1005) and after 6 months of treatment (*N* = 190, 25%) between December 2017 and January 2020. The Addiction Severity Index, PhQ9 and GAD-7 with McNemar-Bowker, and Wilcoxon Signed Rank tests analysis were used to measure patient outcomes across after participation in the program. .

**Results:**

Patients in treatment reported using less heroin (52.1% vs 20.4%), opioids (22.3% vs 11.0%), and alcohol (28.6% vs 13.1%, all *P* < 0.01). Patients reported improved health (53.4% vs. 68.2%, *P* = 0.04), less frequency of disability (8.69 vs. 6.51, *P* = 0.02), symptoms (29.8% vs 21.3%), pain (67.5% to 53.6), worry (45.3% vs 62.3%), anxiety (49.7% vs 23.2%), depression (54.1% vs 23.3%, all *P* < 0.02) after treatment.

**Conclusions:**

This study shows decreased substance use, improved physical and mental health, and reduced symptoms after 6 months of MOUD. Although more research on retention and long-term effects is needed, data shows improved health outcomes after 6 months of MOUD. Lessons learned from implementing this pilot program informed program expansion into other rural areas in need to address some of Colorado’ major public health crises.

**Supplementary Information:**

The online version contains supplementary material available at 10.1186/s13011-021-00424-4.

## Background

Opioid use disorders (OUD) are a national public health crisis due to the rise in both illicit and prescribed opioid use, as well as opioid-related mortality and morbidity [1]. There were almost 50,000 (~ 70% of all drugs) opioid overdose deaths in 2019 [2].

Opioids were involved in 49,860 overdose deaths in 2019 (70.6% of all drug overdose deaths). Opioid use has been shown to associate with comorbid mental health issues, risk behaviors, and economic instability [3, 4], posing a huge burden for people with addictions, their families, and their communities.

The increasing number of cases and overdose deaths has been particularly hard in rural areas that also face disproportionate service gaps [5, 6]. In Colorado, recent reports showed that 1:10 residents live in places with no access to treatment and many in remote rural areas have to travel over 30 miles to seek treatment [6] Although (half million Coloradans reported that they or a love one struggle with prescription pain medications or non-prescribed opioids, 95,000 reported not accessing treatment in 2019 [7]. This underscores the urgency of overcoming access barriers to OUD treatment in remote underserved areas.

Medication for Opioid Use Disorder (MOUD) is an evidence-based practice that combines medication and behavioral interventions [8]. Despite demonstrated efficacy in survival and treatment retention, MOUD services are rarely available in rural areas, leading to inconsistent access to care [9].

Nurses, as one of the largest groups of health service providers in the United States, are in a unique position to care for people with OUD in underserved rural areas [10, 11]. After nurse practitioners became eligible in 2016 to prescribe opioid-based buprenorphine for the treatment of OUD, Colorado Senate Bill 17–074 was introduced as a community-based effort to fund a pilot program bringing Nurse Practitioner (NP) and Physician Assistant (PA)-led MOUD to rural Colorado communities. Between December 2017 and June 2019, the University of Colorado College of Nursing (CU Nursing) worked to engage rural clinics, train providers, and implement a MOUD pilot program in Pueblo and Routt counties. The program added 15 nurse practitioners and physician assistants to the MOUD workforce and served 1005 new patients during the first 18 months [12]. Although the legislation was designed primarily to increase access, CU Nursing and community stakeholders also wanted to know to what extent the program was helping patient’s lives, including mental and physical health, social functioning, employment, disability, and legal system involvement. Here we report patient-centered outcomes after participating in the MOUD program in one of three rural clinics for at least 6 months. Lessons learned from this pilot informed further development of MOUD services in rural Colorado.

## Methods

### Settings and program implementation

The pilot program was designed to implement MOUD at health care sites in two rural counties, Pueblo and Routt, which had OUD-related overdose and death rates higher than state and national averages. Three community clinics received funding between 2017 and 2019: a methadone clinic that added buprenorphine to its services, a community mental health center that expanded its existing MOUD services, and a startup clinic that provided MOUD as part of a multidisciplinary pain services [12]. Service delivery began on December 1, 2017.

### Participants

The clinics in this study serve a racially and ethnically diverse patient population with a mix of health insurance and living experiences. As reported elsewhere [12], patients seeking MOUD services were over 18 years old and met OUD clinical criteria. Prior to starting MOUD, patients underwent a clinical interview by trained staff and consented to receive treatment and to have their clinical information aggregated as part of program evaluation. Patients could refuse providing some information without consequences to their treatment. All procedures were designed to comply with ethical standards for human subject’s studies, although this study was determined by the Colorado Multiple Institutional Review Board to be non-human subject research (protocol #19–2217).

### Procedure and instruments

As part of the program evaluation, MOUD service data were collected between 2017 and 2020. Process data were collected monthly from each of the program sites during the duration of the pilot and for up to 6 additional months for patient follow up. Aggregated agency-level data were analyzed based on monthly reports about services provided, successes, and barriers for implementation and patient retention, and used to answer process evaluation questions [12].

De-identified patient data collected at study entry from 1005 clients included self-reported demographics, medical history, and substance use information. Assessment of treatment outcomes was conducted 6 months after treatment initiation using the validated Addiction Severity Index (ASI), 5th edition [13] to capture past-month drug use, overall health, social functioning, and physical and psychological symptoms. Additional items from the ASI 6th edition [14] were used to evaluate coexisting medical and behavioral health conditions, pain, recent emergency department use or inpatient hospitalization, and other social determinants of health including employment and legal problems. Patients also completed the Patient Health Questionnaire (PHQ-9) to measure depressive symptoms [15] (strong reliability and correlation with substance use, Cronbach’s alpha = 0.90 [16] and the Generalized Anxiety Disorder scale (GAD-7) for anxiety [17], and (IRR = 0.85) Cronbach’s alpha = 0.91 [18]) in substance using populations.

Data were collected at each site by a case manager or intake worker and entered into the REDCap electronic data management system [19] hosted at the University of Colorado Anschutz campus. Aggregated, de-identified data were extracted for analysis and assessed for data integrity at the end of data collection by CU Nursing researchers. The patient-level REDCap data set had a high rate of missing data, which reflects the voluntary nature of the collection and the clinics’ focus on delivering services rather than on data collection, as well as patient loss to follow up.

### Statistical analysis

All statistical tests were conducted on de-identified aggregated data using SPSS version 26 (IBM Corporation). Demographic characteristics were summarized using descriptive statistics, and outcome variables were checked for normal distribution. For continuous variables, mean values and standard errors are presented. Percentages were tabulated for categorical data. Pre-Post changes in client-centered outcome variables were evaluated using McNemar (Y/N) or McNemar Bowker tests for nominal variables, and *t* tests and Wilcoxon Signed Rank Test for normally and non-normally distributed interval-level variables, respectively. Wilcoxon Signed Ranks tests were also performed for confirmatory sensitivity analysis.

## Results

### Patient characteristics

Of 1005 inductions, 288 (28.7%) were still in treatment 6 months after the start of MOUD, and 177 had 6-month substance use data with various degrees of completeness by variable. Complete data on substance use on *evaluable* subjects (those who started treatment and completed *both* the baseline and follow-up assessments, max *n* = 169) were used for the MOUD outcome analysis. Baseline patient characteristics in this study are presented in Table 1. Overall, demographics were similar between patients who remained in treatment for at least 6 months (*evaluable patients*) and those lost to follow-up (*non-evaluable*, also comparable to the whole population) [7]. On average, *evaluable* patients were adults 25 to 44 years old (61.6%), not married (78.9%), White (57.0%) or Hispanic (36.7%), had completed approximately 12.5 years of education (48.5% had completed high school), and had Medicaid insurance coverage (86.9%).

**Table 1:** Baseline demographics of patients in the Colorado MOUD Pilot Program

Characteristic	*Non-Evaluable* ^a^ N (%)	*Evaluable* N (%)	*P*
Gender	*N* = 723	*N* = 174	.21
Female	341 (47.2)	86 (48.6)	
Male	368 (50.9)	87 (49.2)	
Other	1 (0.1)	1 (0.1)	
Race/Ethnicity	*N* = 723	*N* = 177	.33
White non-Hispanic	368 (50.9)	101 (57.0)	
Hispanic	310 (42.9)	65 (36.7)	
Other	24(1.0)	11 (4.0)	
Age	*N* = 714	*N* = 176	.06
18–24 yrs. old	96 (13.3)	19 (10.7)	
25–34 yrs. old	302 (41.8)	69 (39.0)	
35–44 yrs. old	189 (26.1)	40 (22.6)	
45–54 yrs. old	88 (12.2)	28 (15.8)	
55–64 yrs. old	32 (4.4)	17 (9.6)	
Over 65 yrs. old	7 (0.9)	3 (1.7)	
Health Insurance	*N* = 363	*N* = 171	.53
Medicaid only	278 (76.6)	136 (83.4)	
Medicare only	12 (3.3)	10 (6.1)	
Medicaid + Medicare	15 (4.1)	6 (3.5)	
Private	24 (6.6)	8 (4.7)	
Other	7 (1.9)	0 (0)	
None	27 (7.4)	8 (4.5) P	
Employment in the last 3 yrs	*N* = 362	*N* = 168	.37
Full time	101 (27.9)	57 (32.8)	
Part time	71 (19.6)	25 (14.5)	
Unemployed	190 (52.5)	91 (52.6)	
Marital status	*N* = 364	*N* = 171	.77
Married	73 (20.3)	36 (21.0)	
Widowed, Separated, or Divorced	107 (29.4)	58 (33.9)	
Never Married	184 (50.5)	77 (45.0)	
Previous treatment			
No previous OUD treatment	120 (33.1)	47(27.3)	.67
Reason to start treatment^b^	*N* = 355–553	*N* = 167–183	
Self-motivation	453 (82.0)	151(83.1)	.87
Court-order	56 (14.1)	12 (7.1)	***P*** **< .01**
On parole or probation	93 (25.2)	19 (11.1)	***P*** **< .01**

Demographics describe patient characteristics at the start of treatment (n = 190) collected by three participating rural clinical sites. Missing data due to lack of self-reporting varied by item, from ~ 6% for age and race/ethnicity to > 50% for employment or substance use specification; actual counts per variable are noted

^a^ *Evaluable* patients are those who started the MOUD treatment and completed both baseline and follow-up surveys, as contrast with *non-evaluable* patients had an induction but withdrew or were lost to follow-up and thus did not have follow-up data regarding substance use. ^b^ Reasons for start treatment are all independent variables and do not add up to 100%

**Fig. 1:**
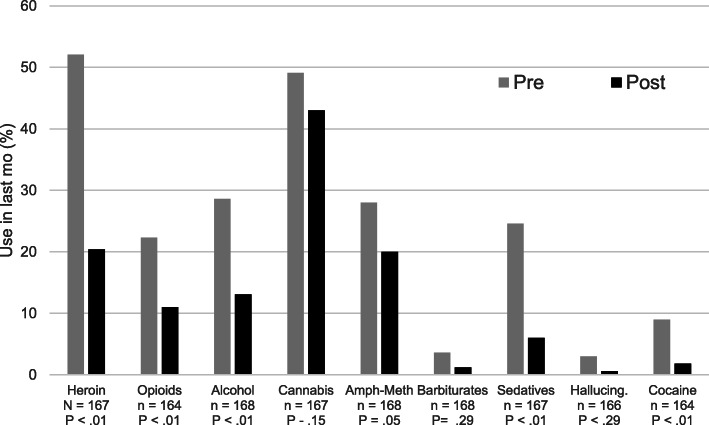
Changes in substance use after participation in the Colorado MOUD Pilot Program. Figure shows the percentage of patients reporting *any* day of use of the indicated substances in the previous month, both at baseline (pre) and after 6 months of treatment (post), with *P* values for changes from baseline (Mc Nemar-Bowker; < .05 = statistically significant). Aggregated data from patients (*n* = 168) in 3 rural sites participating in the MOUD program is shown. Missing data due to lack of self-reporting varied by item and actual counts per paired variable are noted. Amph-Meth = Amphetamine – methamphetamine.

About half of the patients had been unemployed during the last 3 years (52.6%), with no differences between *evaluable* and *non-evaluable* patients. About a third of patients presented to the clinic based on a judicial system referral or were worried about legal problems (30%). About 1 in 3 patients reported having had some outpatient (32.0%) or inpatient detoxification (27.3%) treatment previously, and 2.37% had not attempted any other treatment methods in the past, with no differences between *evaluable* or *non-evaluable* patients. Most patients (83.1%) initially reported that their current decision to get treatment was self-prompted. However, compared to patients who started but were lost to follow up, evaluable patients were less likely to be court-ordered (14.1% vs 7.1%, *P =* 0.01) or to be on parole or probation (25.2% vs 11.1%, *P =* 0.01). All patients had a history of use opioids and/or heroin, and many also used other substances (32–38%); most were in poor to moderate health (80.2%) and suffered moderate to extreme pain (65%, see Fig. 1).

### Changes in clinical outcomes after MOUD treatment

Pre–post treatment changes on measures regarding substance use were assessed among the 169 *evaluable* patients with complete baseline and 6-month data on substance use (Fig. 1). Those who were still in treatment reported less heroin use in the past month than at start, with 52.1% vs 20.4% of patients reporting *any* heroin use, and 37.1% vs 5.4% using heroin *daily.* A Wilcoxon Signed-Rank Test indicated that the median post-test ranks were statistically different than the pre-test ranks, Median 1.0 (IQR 30.00), vs 0.0 (IQR 20.00) (Supplemental Table 1), all *P* < .01. They also reported less use of prescription opioids (22.3% vs 11.0% of patients reported *any* opioid use, with 11.7% vs 3.5 using opioids daily, *P <* .01, and a reduction in the number of days, *P =* .021). After 6 months, patients also reported significantly less alcohol use (28.6% vs 13.1% of patients reported *any* drinking), with 4.40% of participants who drank *daily* at baseline vs 0.6% post treatment. Patients also reported drinking less days per month (Median 0.0 vs 0.0 vs days, *P* < .001). Interestingly, use of cannabis increased significantly (19.1 to 30.2% of *daily* use, *P* < .02, Median number of days 0.0 vs 0.0, *P =* .028), although the percent of people reporting *any* use did not change (49.1% vs 43.0%), which could indicate a compensatory pain management strategy given that 41% of patients at baseline had indicated marijuana use for pain management. The sensitivity analysis for substance use (Supplemental Table 1) yielded similar trends in terms of both significance and directionality. Patients reported overall using less sedatives (26% vs 6.0%), cocaine (9.0% vs 1.8%) both *P* < .01), and very small reduction methamphetamine or barbiturates*any day* use after treatment (*P* < .05).

**Fig. 2:**
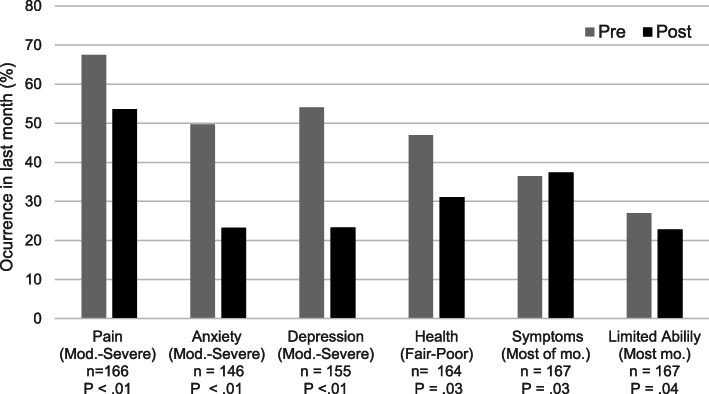
Changes in Physical and Mental Health after MOUD treatment. Figure shows the percentage of patients reporting the following health related issues during the past month, at (pre) and after 6 months of treatment (post), with *P* values for overall changes from baseline: Pain = moderate to severe pain or discomfort; Anxiety (GAD-7 scores for moderate to severe anxiety); Depression (PHQ-9 scores corresponding to moderate to severe depression); Poor Health (less than good health or poor to fair); Symptoms (physical or medical symptoms over half of the month); Limited ability (unable to carry out normal activities because of physical or mental symptoms during over half of the month). *P* values shown changes from baseline (Mc Nemar-Bowker; < .05 = statistically significant, *evaluable* data, n = 167). Missing data due to lack of self-reporting varied by item and actual counts per paired variable are noted.

Health changes after 6 months of treatment are shown in Fig. 2. The percent of patients reporting moderate to severe pain or discomfort diminished significantly after treatment (67.5 to 53.6%, *P* < .01). Patients also showed significant reduction in anxiety (49.7% at baseline vs 23.2% post treatment showed moderate to high anxiety), based on GAD-7 average scores decreasing significantly after treatment (10.5 ± 7.4 vs 6.1 ± 5.4, *P <* .01), which corresponds to a shift in clinical interpretation from “moderate anxiety” to “mild anxiety.” Major depressive levels paralleled this trend (54.1% vs 23.3% of patients scored moderate to high depression, *P <* .01), with an average clinical shift from “moderate depression” to “mild depression” (scores: 10.5 ± 7.15 vs 6.2 ± 5.83, *P <* .01). The frequency of symptoms slightly but significantly changed (daily: 29.8% vs 21.3%; no symptoms: 35.4% vs 44.8%; overall change *P* = .03). There were fewer patients reporting they were unable to carry out normal activities because of physical or medical problems after treatment for *most days* in the month after treatment (26.9% vs. 22.7%, *P* = .04) or *any day* in the month 53.3% vs 43.1%, *P* = 0.04). 3). Changes in scores were confirmed with sensitivity analyses (*P <* .01 for the nonparametric tests).

### MOUD treatment and changes in social determinants of health

**Fig. 3:**
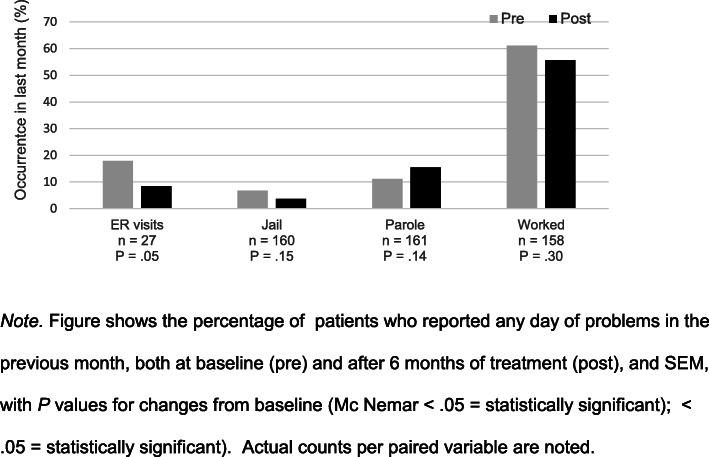
Change in social issues and services among Colorado Pilot MOUD Program patients.. *Note*. Figure shows the percentage of patients who reported any day of problems in the previous month, both at baseline (pre) and after 6 months of treatment (post), and SEM, with *P* values for changes from baseline (Mc Nemar < .05 = statistically significant); < .05 = statistically significant). Actual counts per paired variable are noted

Baseline data on factors associated with OUD, including employment, health care utilization, and legal system involvement, demonstrated that the program reached a patient population with substantial life problems related to OUD. After 6 months of MAT, patients had slightly but not significantly fewer emergency room visits in the prior month, *P =* .10 (Fig. 3). Likewise, there were no changes for the patients who reported spending days in jail on the previous month, *P =* .89, or being on parole, although those numbers were very low at the start of treatment, leaving limited room for improvement. There were no changes in the percentage of patients who worked on the previous month (61% vs 56%, *P* = 0.29) or the number of days worked .

**Fig. 4:**
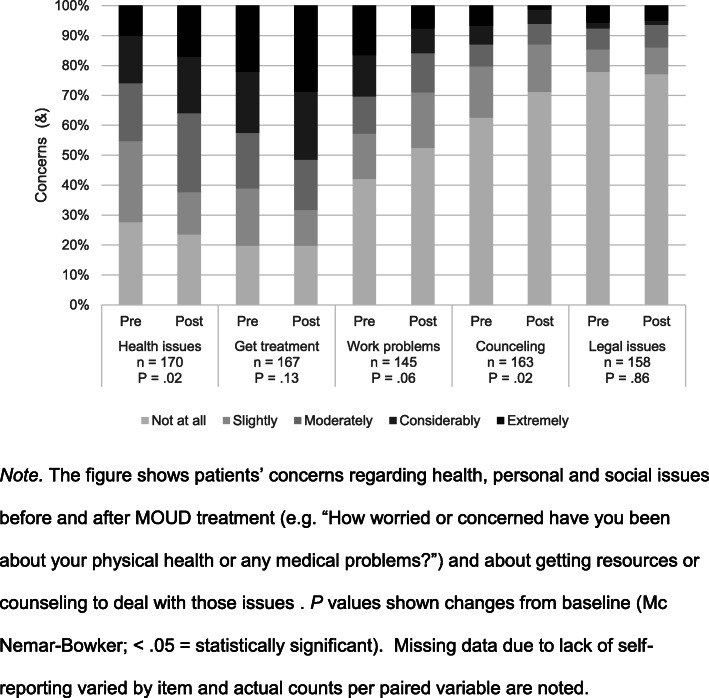
Changes in patient perceptions regarding physical health or social concerns. *Note*. The figure shows patients' concerns regarding health, personal and social issues before and after MOUD treatment (e.g. “How worried or concerned have you been about your physical health or any medical problems?”) and about getting resources or counseling to deal with those issues. *P* values shown changes from baseline (Mc Nemar-Bowker; < .05 = statistically significant). Missing data due to lack of self-reporting varied by item and actual counts per paired variable are noted

Fig. 4 shows patients’ perceptions of their health and social issues from baseline to 6 months after treatment start. After 6 months, patient concerns regarding their overall health increased after treatment, going from 45.3 to 62.3%, moderately to extremely concerned (*P =* .02); however, their perception of the importance of continuing treatment did not have a significant change (61.1% vs 68.1% reporting at least moderate importance, *P =* .12). On the other hand, patients seemed less concerned about work issues after treatment (overall 43.6% vs 29.0% were concerned, *P =* .06) or the need for work-related counseling (drop from 20.2 to 12.9% in moderately to highly concerned, *P =* .02). There were no significant changes in concerns about legal issues.

### Retention in care

Monthly reporting and community of practice debriefs revealed several efforts dedicated to program implementation, as well as challenges to patient retention in care. As reported in detail elsewhere [12], clinicals sites engaged community partnerships which were essential to recruit patients However, this take significant effort to develop, and despite outreach efforts (partnership with jails, hospitals, churches, local health care providers), stigma regarding substance use and associated mental health issues remains major treatment barriers in rural areas and requires strategies to build support for treating patients and a main focus for program continuation.

Retaining patient in care was a major problem faced by participating clinics, although clinics attempted to partially address it by using multi-modal treatment that includes behavioral and supportive interventions as well as medication. Likewise, clinical sites revealed main challenges to track patients who drop out of treatment, and dealing with mobile populations, homeless, although some cycling in and out of care seems to be normal, and having an open-door policy to welcome back patients who want to resume treatment seems essential, and discussed the need to dedicate resources to maintain a patient registry and to track individual patients who fall out of care. Reimbursement is another challenge clinics faced, and may take many months to develop properly, underscoring the need for grant funding before MOUD can become self-sustaining through fee for service income. Lessons learned for ongoing barriers and facilitators encountered during implementation informed the development of policy to practice of new MAT/MOUD programs.

## Discussion

There is an urgent need to expand access to OUD treatment in rural areas. This program provided access to MOUD treatment to 1005 patients in two rural counties. Our analysis showed that patients who were still in treatment after 6 months had significant reductions in opioid use, alcohol use, and other drug use, as well as improvements in health and employment indicators and clinically meaningful decreases in both anxiety and depression symptoms. These changes have real-life consequences for patients, families, and communities.

### Social determinants of health and OUD

Our data showed that the MOUD services reached a rural patient population with significant life problems related to OUD. A high rate of unemployment was reported among MOUD treatment recipients, ~ 25-fold higher than the average state population [20]. Unemployment has been associated with poorer health outcomes, including greater psychological distress [21] and food insecurity [22], which in turn is linked to adverse mental health outcomes, including suicide [23, 24]. People seeking OUD services presented with moderate mental health issues, physical symptoms, and rates of ER utilization, which is characteristic for this population. OUD is also associated with high rates of medical comorbidities, including HIV risk and Hepatitis C infection [25, 26]. Because comorbidities can associate to early death in individuals with OUD [26], it is imperative to address them through comprehensive patient care approaches.

### Treatment outcomes on opioid and other substance use

Our findings add to previous data showing the efficacy of MOUD, which involves the combination of behavioral therapies with FDA-approved medications as the standard of care for OUD. Treatment offered by clinic sites included methadone delivered by a regulated opioid treatment program as well as buprenorphine and naltrexone used by other outpatient clinics [12]. Consistent with the literature [9], patients in our study who completed > 6 months of MOUD treatment used opioids and other substances on fewer days, had less health care utilization, and had improved self-reported health compared to their own pre-treatment levels. Alcohol use was also prevalent in this population, especially in combination with other substances. MAT is also a valid treatment for alcohol use disorder [27], and MOUD treatment also had a positive impact on the use of alcohol and sedative analgesics. In contrast, daily usage of marijuana seemed to have increased after 6 months of MOUD treatment. Although outside the scope of this study, patients may use marijuana to cope with pain, anxiety, or other concerns. This finding suggests the importance of screening for cannabis use and underlying reasons for use [28]. A shift in marijuana use during OUD treatment might reveal new challenges due to Colorado’s legalization of marijuana [29].

### Health outcomes and other opioid related crises

Patients’ health status improved after 6 months of MOUD treatment. Overall, patients reported less concern about their health, but also placed more importance on receiving treatment for health concerns, which might suggest increased motivation for self-management and health maintenance. There also was a significant reduction in patients’ concerns about pain, which is a very positive sign. Our findings align with the National Academy of Medicine’s recommendations regarding the need for rigorous pain management strategies and tapering doses for enhanced outcomes [30, 31]. Treatment with MOUD drugs has been also shown to decrease OUD-related mortality [32]. Although out of the scope of this study, reductions in substance use has been associated to to fewer opioid overdoses, long-term enhanced quality of life, and reduced mortality among patients who remain in treatment for at least 6 months [33]. While small numbers preclude reaching major conclusions, opioid-related deaths in these two counties indeed dropped from 2017 to 2019 (18.0 vs 14.8 and 20.6 vs no cases per 100,000 people in Pueblo and Routt, respectively) [34].

Chronic pain affects many aspects of a person’s daily life, including physical and mental health, family, work, and social relationships [35]. Opioid use can also cause trouble concentrating, maintaining sleep, and depression [36] and pilot Colorado MOUD patients reported high levels of anxiety and depression at the start of treatment. Similar to previous studies, our data demonstrated significant decreases in both anxiety and depression after MOUD treatment. Additionally, because an estimated 20% of opioid overdose deaths are related to suicide, our results underscore the importance of thorough screening and prompt treatment for mental health concerns [37]. Study sites offered a range of behavioral health treatments [12] which are also linked to improved patient outcomes independent of the type of psychosocial modality offered.

Impaired social functioning and inability to successfully fulfill personal or societal roles are debilitating features of OUD and hallmark criteria for diagnosis [38], Notably, patients who completed 6 months of MOUD treatment also reported an improved ability to function during the prior month. While there were no significant changes in the number of days of paid work, which could reflect in loss of productivity resulting from pain and OUD, patients reported fewer concerns with work related-problems.

### Access versus retention in care

Pilot program grantees advertised their services to prospective OUD patients through partnerships with medical providers in the community, law enforcement agencies, local orgRich RJ, Chou R, Mariano ER, et al. Best Practices, Research Gaps, andanizations, churches, and community groups [12]. Engagement in MOUD treatment has been associated with better patient retention than other OUD treatments [32], superior outcomes, and less relapse [26, 39]. Retention in treatment varies across setting [40]. Although high retention could be achieved at 3 months in academic settings [41], the Colorado MOUD pilot program in rural community clinics had low retention at 6 month, which could be a difference in time of assessment, model of care, study design and setting. While we did not assess the reason for patients leaving the program, published literature suggests that loss to follow up could be due to patients “feeling cured” or having lingering opioid cravings or ongoing pain [39, 42]. Consistent care coordination and a behavioral health component of MOUD is key for retention because it helps patients actively manage their OUD [41, 43]. Equipping providers with modalities like motivational interviewing or mindfulness has proven successful for a variety of conditions ranging from chronic pain management to mental health [44] and has the potential to enhance patient retention.

Societal influences are another potential cause of patient dropout. Despite available treatment, people with mental disorders commonly fail to seek help due to the stigma associated with these services [45]. Our results showed that more clients who dropped out were court-ordered to start treatment. Although there were no differences in self-reported motivation at the start of treatment, patients who continued might have a combination of true self-determination, commitment to change, and external support. Further studies should explore motivation and the role of social determinants of health and support systems in predicting whether OUD patents remain in treatment. Despite community outreach efforts, opioid-related stigma and negative attitudes are still prevalent, and some still favor withdrawal and detox modalities despite the detrimental effects of these treatment approaches on care retention. Our stakeholder-informed evaluation confirmed that people with OUD are widely stigmatized and stereotyped (unpublished work) which can affect patient functioning [46] and whether patients seek and continue treatment [10, 12]. These results emphasize a need for ongoing efforts to lower stigma, maintain open door policies for returning to treatment, and resources to maintain a patient registry and to track individuals who fall out of care, given the myriad of factors that contribute to patient success.

### Study limitations

Several limitations are noted. First, we used subjective assessments, which may not be reliable if patients were under detox and having withdrawal symptoms. However, the measures used are similar to accepted processes used in other studies. Second, results are limited to patients still in treatment and providing data. Not all patients lost to follow-up were necessarily treatment failures; some simply may be missing data, which could be partly attributed to voluntary data collection (i.e., clinics not tracking their patients well over time vs patients not returning for care). Because the *evaluable* patients were demographically and clinically similar to the *non-evaluable* ones at the start of treatment, the risk of biased results is minimized. Third, the evaluation focused on data from only two rural counties that may not represent other parts of Colorado or of the country. Finally, this study lacked a comparison group, therefore causality cannot be inferred. Likewise, competing explanations like history or maturation effects cannot be ruled out. Moreover, people remaining in treatment might have a stronger motivation at baseline, and those leaving could perceive the treatment was not effective for them, all of which should be further assessed. Our results demonstrate positive outcomes for those patients with *evaluable* data (remaining in treatment), although we cannot infer about reasons why people remain or drop treatment. As public health impact is limited by retention in care, this should be a primary focus of future studies.

## Conclusions

The ongoing opioid crisis necessitate heightened efforts to implement evidence-based care across the United States. Health care providers and patients in rural and small towns have limited resources, and both behavioral and social barriers can impede treatment success. The results of this study were part of a multi-pronged statewide effort to address the opioid crisis in both metropolitan and rural, underserved areas. Results also informed expansion efforts to improve the lives of Coloradans with OUD. While more research is needed regarding retention and long-term effects, this study highlights the effectiveness of MOUD as one strategy to address this major public health crisis.

## Supplementary Information

**Additional file 1: Supplemental Table 1.** Change in substance use from baseline (pre) to 6-months (post) of MOUD treatment.

## Data Availability

Due to the nature of sensitive information, the datasets used in this study are available from the corresponding author and with stakeholder’s permission upon reasonable request.

## Abbreviations

ASI: Addiction severity index (ASI)
CU Nursing: University of Colorado College of Nursing
OUD: Opioid use disorders
MOUD: Medication for substance use disorders
MAT: Medication assisted treatment
PHQ-9: Patient health questionnaire (depressive symptoms)
GAD-7: The generalized anxiety disorder scale
SPSS: Statistical package for the social sciences
