# Supplementary material for: Outcomes from the medication assisted treatment pilot program for adults with opioid use disorders in rural Colorado
Source: Subst Abuse Treat Prev Policy. 2022 Jan 3;17:1. doi: 10.1186/s13011-021-00424-4 (PMC8722086; doi:10.1186/s13011-021-00424-4)
Supplement: Supplementary file 1 — Additional file 1: Supplemental Table 1. Change in substance use from baseline (pre) to 6-months (post) of MOUD treatment. [file 13011_2021_424_MOESM1_ESM.docx]

**Supplemental Table 1.** Change in substance use from baseline (pre) to 6-months (post) of MOUD treatment.

|  | *Pre* | *Post* |  | *P* |  |
| --- | --- | --- | --- | --- | --- |
| Heroin Use in the past mo. (N = 167) ^a^ |  |  |  |  |  |
| Days of use (mean, SDV) ^b^ | 13.06 (14.27 | 3.68 (8.65) |  | **<.001** |  |
| Days of use (median, IQR)^c^ | 1.00 (30.00) | 0.00 (0.00) |  | **<.001** |  |
| Any use (%)^d^ | 52.10 | 20.40 |  | **<.001** |  |
| Daily use (%)^e^ | 37.10 | 5.40 |  | **<.001** |  |
| Opioids Use in the past mo. (N = 164) |  |  |  |  |  |
| Days of use (mean, SDV) | 3.68 (9.1) | 1.86 (6.52) |  | **.029** |  |
| Days of use (median, IQR) | 0.00 (0.00) | 0.00 (0.00) |  | **<.001** |  |
| Any use (%) | 22.30 | 11.00 |  | **<.001** |  |
| Daily use (%) | 11.67 | 3.45 |  | **<.001** |  |
| Alcohol Use in the past mo. (N = 168) |  |  |  |  |  |
| Days of use (mean, SDV) | 3.13 (7.49) | 1.27 (4.72) |  | **<.001** |  |
| Days of use (median *(IQR*) | .00 (0.00) | .00 (0.00) |  | **<.001** |  |
| Any use (%) | 28.60 | 13.10 |  | **<.001** |  |
| Daily use (%) | 4.40 | 0.60 |  | **<.001** |  |
| Cannabis Use in the past mo. (N = 167) |  |  |  |  |  |
| Days of use (mean, SDV) | 8.78 (12.17) | 11.02 (13.89) |  | .013 |  |
| Days of use (median, IQR) | 0.00 (20.00) | 0.00 (30.00) |  | **.041** |  |
| Any use (%) | 49.10 | 43.00 |  | .150 |  |
| Daily use %) | 19.60 | 31.80 |  | **<.001** |  |
| Amphetamines in the past mo. (N = 168) |  |  |  |  |  |
| Days of use (mean, SDV) | 3.23 (7.23) | 3.30 (8.27) |  | .918 |  |
| Days of use (median, IQR) | .00 (.00) | .00 (0) |  | .729 |  |
| Any use (%) | 28.00 | 20.00 |  | **.047** |  |
| Daily Use (%) | 3.60 | 6.00 |  | .388 |  |
| Barbiturates Use in the past mo. (N = 168) |  |  |  |  |  |
| Days of use (mean, SDV) | .28 (2.40) | .01 (.11) |  | .159 |  |
| Days of use (median *(IQR*) | .00 (2.00) | .00 (0.00) |  | **.049** |  |
| Any use (%) | 3.60 | 1.20 |  | .289 |  |
| Daily use (%) | .60 | .00 |  | .290 |  |
| Sedatives Use in the past mo. (N = 167) |  |  |  |  |  |
| Days of use (mean, SDV) | 2.59 (6.75) | 1.11 (5.52) | <.01 | **.001** |  |
| Days of use (median *(IQR*) | .00 (0.0) | .00 (0.0) |  | **<.001** |  |
| Any use (%) | 24.6 | 6.0 |  | **<.001** |  |
| Daily use (%) | 3.6 | 3.0 |  | 1.000 |  |
| Hallucinogens Use in past mo. (N = 166) |  |  |  |  |  |
| Days of use (mean, SDV) | .048 (0.29) | .12 (1.55) |  | .109 |  |
| Days of use (median *(IQR*) | .00 (0.0) | .00 (0.0) |  | .524 |  |
| Any use (%) | 3.00 | .6 |  | .125 |  |
| Daily use (%) | .00 | .00 |  |  |  |
| Cocaine Use in the past mo. (N = 167) |  |  |  |  |  |
| Days of use (mean, SDV) | .319 (1.80) | .24 (2.45) |  | .759 |  |
| Days of use (median, IQR) | .00 (.00) | .00 (0.00) |  | **.032** |  |
| Any use (%) | 9.0 | 1.8 |  | **.004** |  |
| Daily use (%) | .0 | .0 |  |  |  |

*Notes*. This table displays the changes in substance use in adults participating in MOUD.

^a^ Numeric variables were summarized by Means (SDV) for comparison

^b^ Numeric variables were summarized by Median (IQR) and pre-post Wilcoxon Signed-Rank Test *P* value

## ^c^ Any use for categorical variables were summarized by the % in each category and paired pre-post McNemar *P* value (Y/N).

## ^d^ Daily use is shown as a measure from categorical analysis of the month (no use, less <15 days, over 15 days, every day); as the % in each category and McNemar-Bowker pre-post p-value (multiple categories).

## ^d^ The number of subjects in the total sample with longitudinal evaluable data is reported next to each variable’s name.
